# Supplementary material for: Orchestrated Response of Intracellular Zwitterionic Metabolites in Stress Adaptation of the Halophilic Heterotrophic Bacterium Pelagibaca bermudensis
Source: Mar Drugs. 2022 Nov 19;20(11):727. doi: 10.3390/md20110727 (PMC9695272; doi:10.3390/md20110727)
Supplement: Supplementary file 1 [file marinedrugs-20-00727-s001.zip › marinedrugs-1966440-supplementary.pdf]

marine drugs

Supplementary Materials

Orchestrated Response of Intracellular Zwitterionic Metabolites in Stress Adaptation of the Halophilic Heterotrophic Bacterium *Pelagibaca bermudensis*

Muhaiminatul Azizah [1] and Georg Pohnert [1,2,*]

1 Bioorganic Analytics, Institute for Inorganic and Analytical Chemistry, Friedrich Schiller University, Lessingstrasse 8, D-07743 Jena, Germany
2 MPG Fellow Group, Max Planck Institute for Chemical Ecology, Hans-Knöll-Straße 8, D-07745 Jena, Germany
* Correspondence: georg.pohnert@uni-jena.de

Contents:

Figure S1.1 – S1.2 Chromatographic profile of zwitterionic metabolites from *P. bermudensis* using UHPLC with detection by ESI-HRMS.
Figure S2.1. Comparison of MS/MS spectra of DMSP from *P. bermudensis* and reference standard
Figure S2.2. Comparison of MS/MS spectra of DMSOP from *P. bermudensis* and reference standard
Figure S2.3. Comparison of MS/MS spectra of cysteinolic acid from *P. bermudensis* and reference standard
Figure S2.4. Comparison of MS/MS spectra of gonyol from *P. bermudensis* and reference standard
Figure S2.5. Comparison of MS/MS spectra of glycine betaine (GBT) from *P. bermudensis* and reference standard
Figure S2.6. Comparison of MS/MS spectra of carnitine from *P. bermudensis* and reference standard
Figure S2.7. Comparison of MS/MS spectra of ectoine from *P. bermudensis* and reference standard
Figure S2.8. Comparison of MS/MS spectra of choline from *P. bermudensis* and reference standard
Figure S2.9. Comparison of MS/MS spectra of 4-hydroxyproline from *P. bermudensis* and reference standard
Figure S2.10. Comparison of MS/MS spectra of proline from *P. bermudensis* and reference standard
Figure S2.11. Comparison of MS/MS spectra of sarcosine from *P. bermudensis* and reference standard
Figure S3.1. OD_{600} during the growth curve of *P. bermudensis* cultures grown in marine broth with NaCl concentration of 0.33 M – 1.33 M
Figure S3.2. $OD6_{00}$ during the growth curve of *P. bermudensis* cultures grown in marine broth with NaCl concentration of 0.033 M – 0.33 M and the absence of NaCl
Figure S3.3. OD_{600} during the growth curve of *P. bermudensis* cultures grown in marine broth with a temperature range of 18℃ - 38℃; error bars represent standard deviation of three biological replicates.
Figure S3.4. OD_{600} during the growth curve of *P. bermudensis* cultures grown in marine broth under incubation with extract of *T. striata*; error bars represent standard deviation of three biological replicates
Figure S4. Cell densities of *P. bermudensis* under different stress treatments.
Figure S5. The intracellular concentration of sulfur- and nitrogen-containing zwitterionic metabolites in *P. bermudensis* HTCC2601 after treatment with *T. striata* extracts in different concentrations.
Table S1. Concentration of sulfur- and nitrogen-containing zwitterionic metabolites from *T. striata* by LC-HRMS

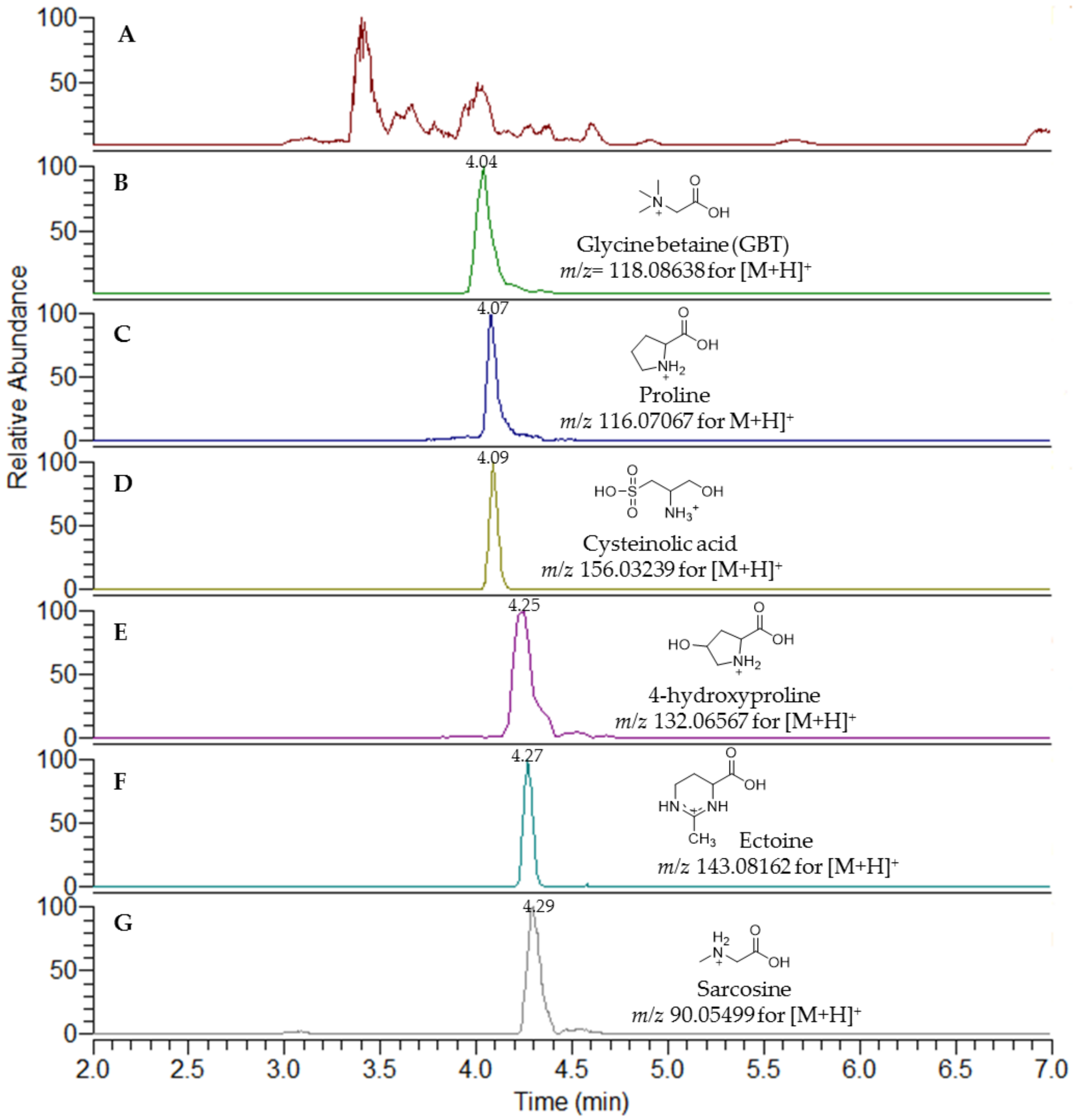

Figure S1.1 Chromatographic profile of zwitterionic metabolites from *P. bermudensis* using UHPLC with detection by ESI-HRMS. (**A**) Total Ion Count (TIC), (**B**) GBT ion trace *m*/*z* 118.08638, (**C**) proline ion trace *m*/*z* 116.07067, (**D**) Cysteinolic acid ion trace *m*/*z* 156.03239, (**E**) 4-hydroxyproline ion trace *m*/*z* 132.06567 (**F**) ectoine ion trace *m*/*z* 143.08162, (**G**) Sarcosine ion trace *m*/*z* 90.05499.

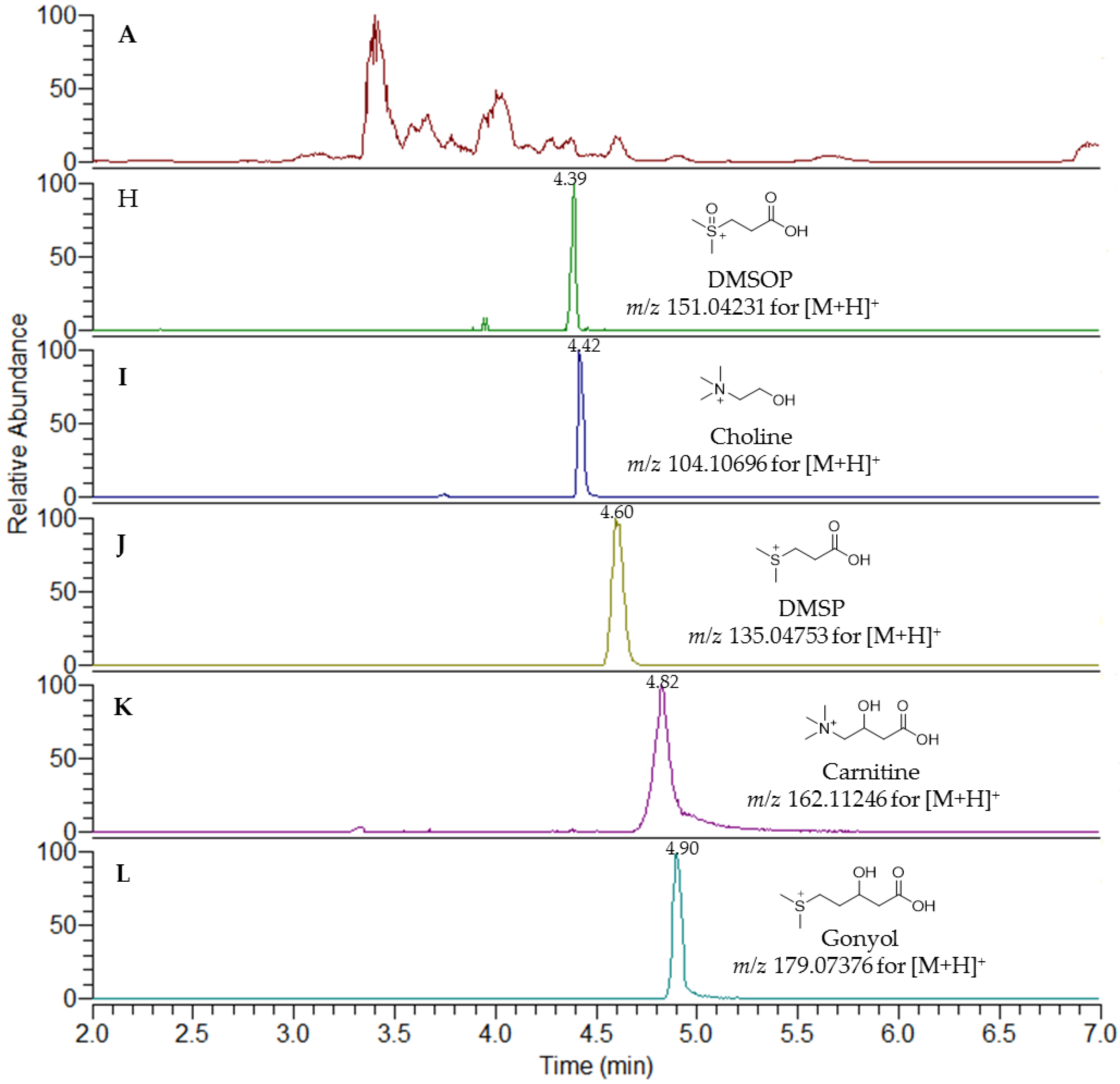

Figure S1.2 Chromatographic profile of zwitterionic metabolites from *P. bermudensis* using UHPLC with detection by ESI-HRMS. (**A**) Total Ion Count (TIC), (**H**) DMSOP ion trace *m*/*z* 151.04231, (**I**) Choline ion trace *m*/*z* 104.10696, (**J**) DMSP ion trace *m*/*z* 135.04753, (**K**) carnitine ion trace *m*/*z* 162.11246 (**L**) Gonyol ion trace *m*/*z* 179.07376.

Comparison of MS/MS spectra of zwitterionic metabolites from *P. bermudensis* and reference standards

All MS/MS spectra of our reference standards were deposited and publicly available online in the GNPS spectral libraries. The charts were made using the Metabolomics Spectrum Resolver (https://metabolomics-usi.ucsd.edu/) [1] and scannable QR-codes link out to the individual annotated charts and library spectra with their metadata. All spectral mirror charts had cosine similarity scores not lesser than 0.98. Identical MS/MS spectra proved the identity of compounds from *P. bermudensis* were identical compounds with reference standards.

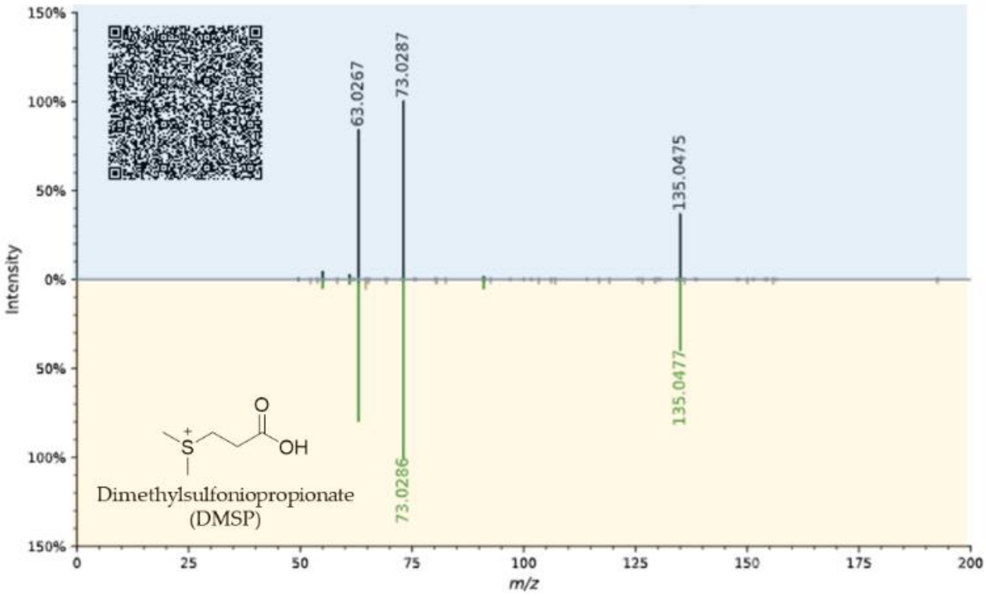

Figure S2.1. Comparison of MS/MS spectra of DMSP from *P. bermudensis* (top plot) and reference standard (bottom plot). Link could be found here, CCMSLIB00006716179

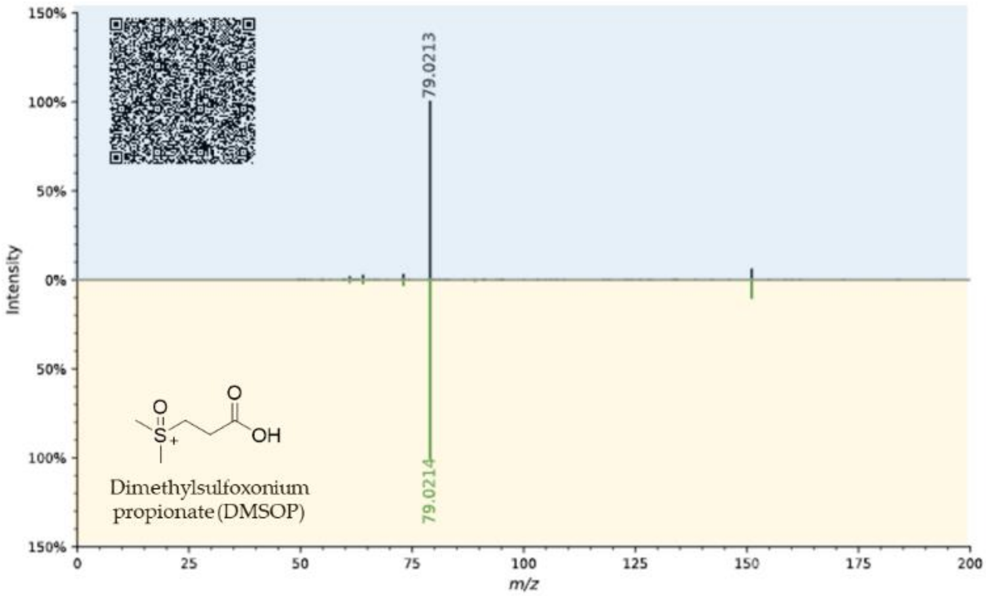

Figure S2.2. Comparison of MS/MS spectra of DMSOP from *P. bermudensis* (top plot) and reference standard (bottom plot). Link could be found here, CCMSLIB00006716180

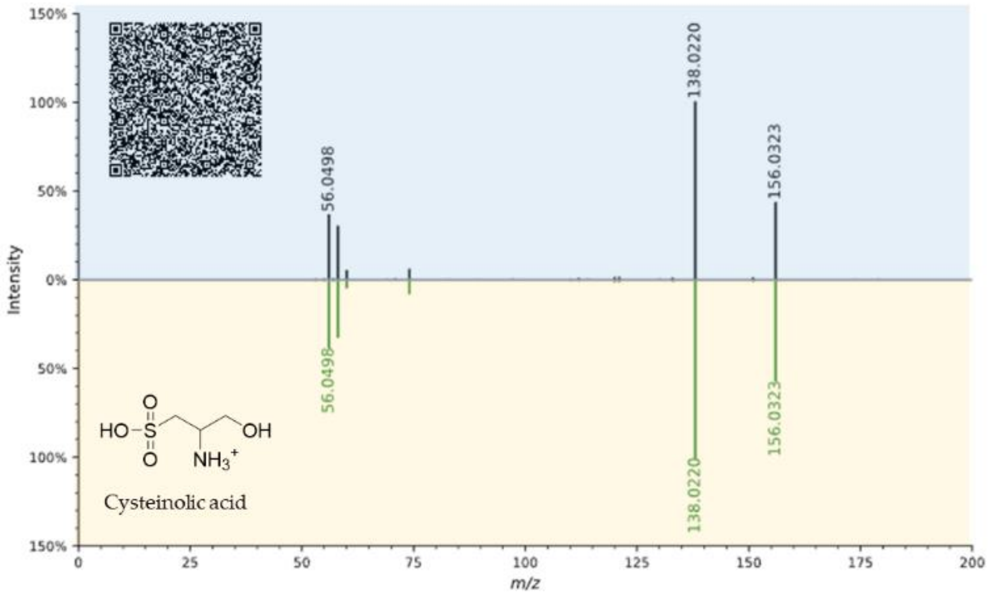

Figure S2.3. Comparison of MS/MS spectra of cysteinolic acid from *P. bermudensis* (top plot) and reference standard (bottom plot). Link could be found here, CCMSLIB00008851455

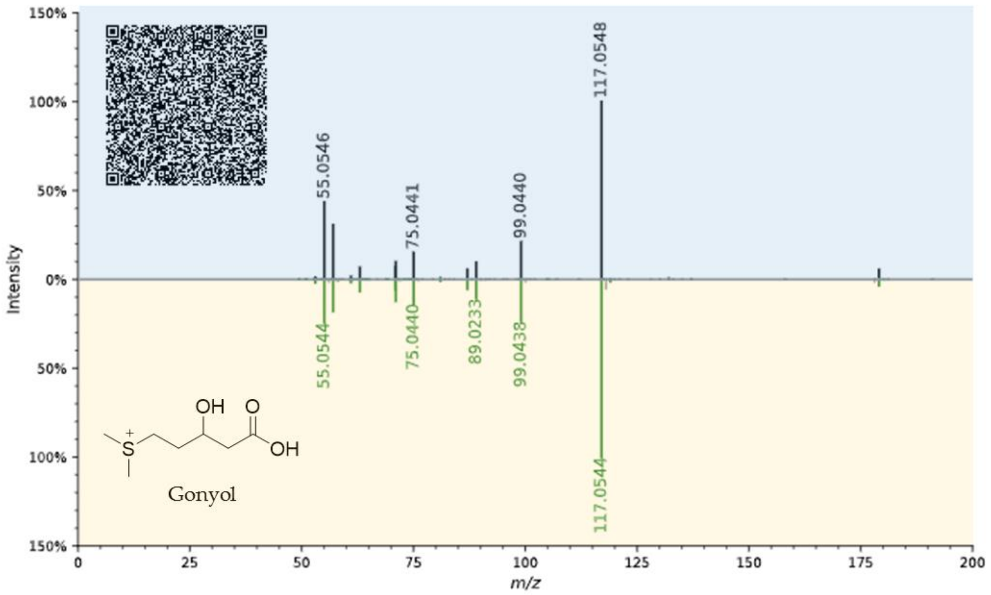

Figure S2.4. Comparison of MS/MS spectra of gonyol from *P. bermudensis* (top plot) and reference standard (bottom plot). Link could be found here, CCMSLIB00006716181

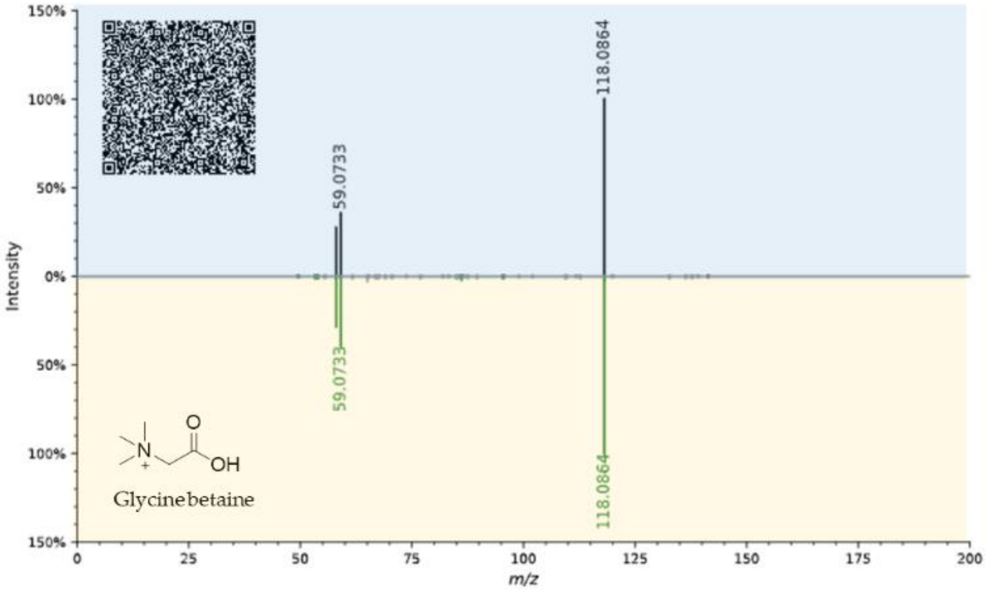

Figure S2.5. Comparison of MS/MS spectra of glycine betaine (GBT) from *P. bermudensis* (top plot) and reference standard (bottom plot). Link could be found here, CCMSLIB00006716183

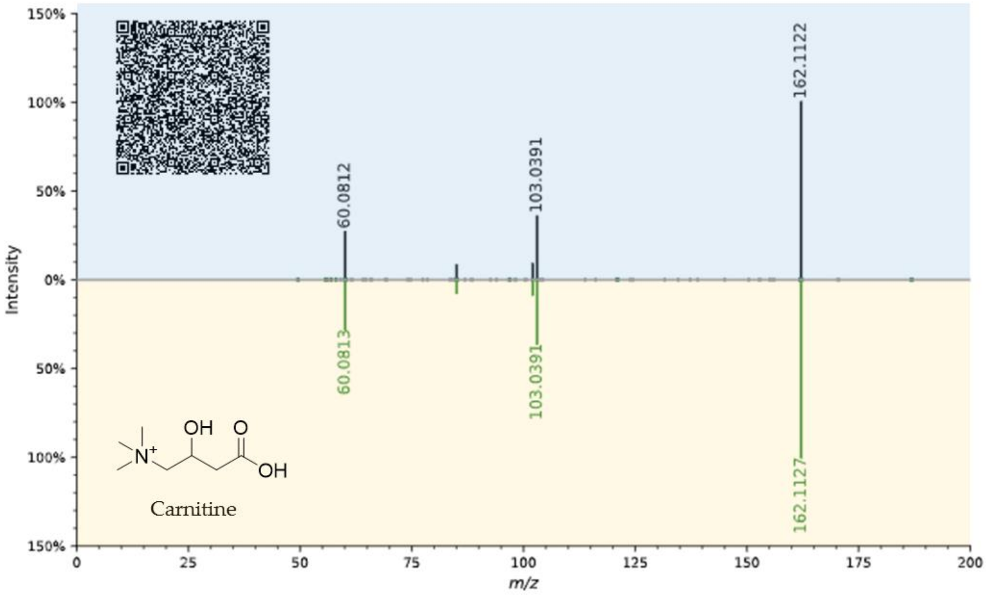

Figure S2.6. Comparison of MS/MS spectra of carnitine from *P. bermudensis* (top plot) and reference standard (bottom plot). Link could be found here, CCMSLIB00008851519

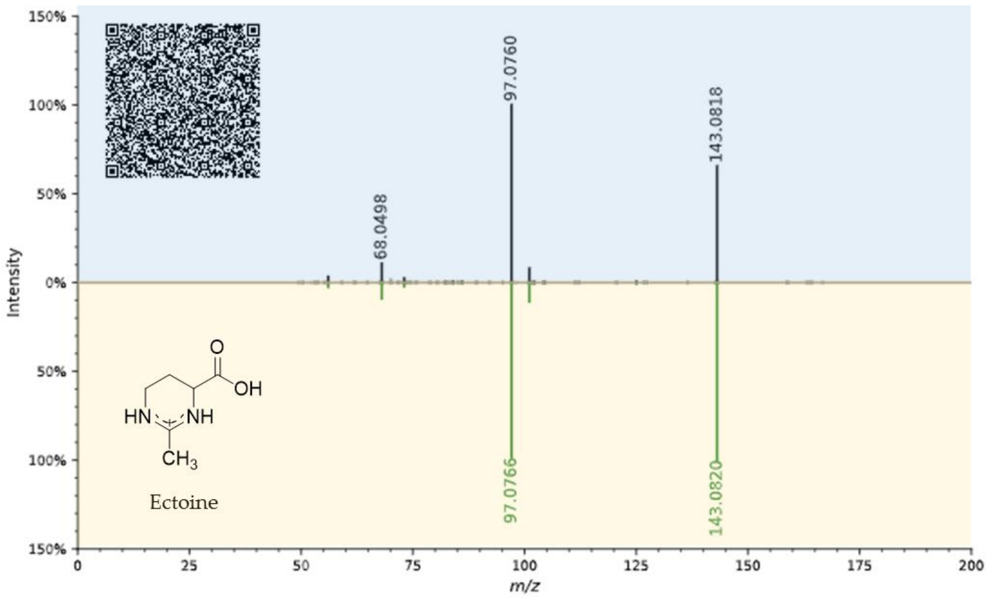

Figure S2.7. Comparison of MS/MS spectra of ectoine from *P. bermudensis* (top plot) and reference standard (bottom plot). Link could be found here, CCMSLIB00006717988

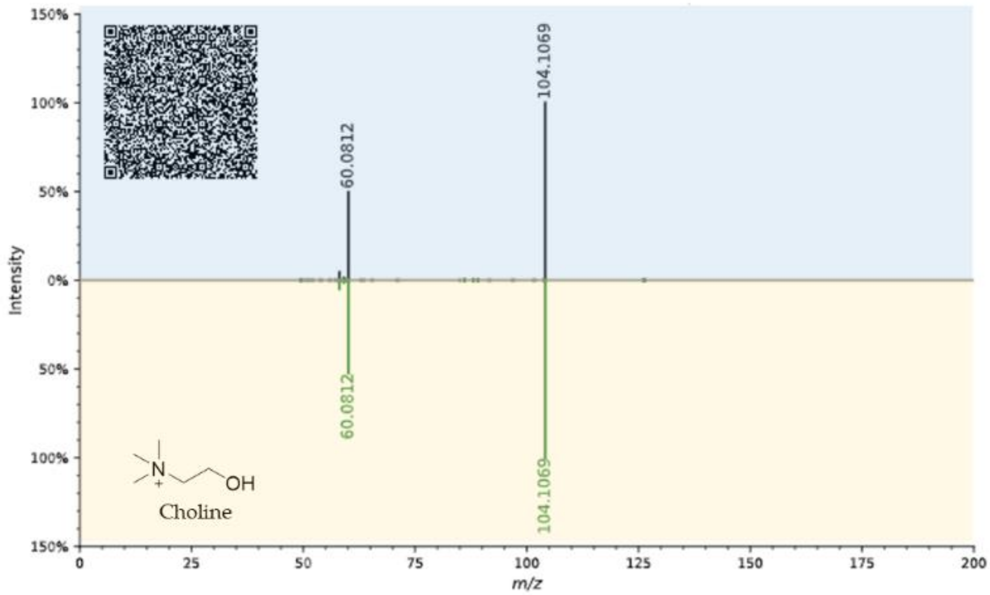

Figure S2.8. Comparison of MS/MS spectra of choline from *P. bermudensis* (top plot) and reference standard (bottom plot). Link could be found here, CCMSLIB00008851521

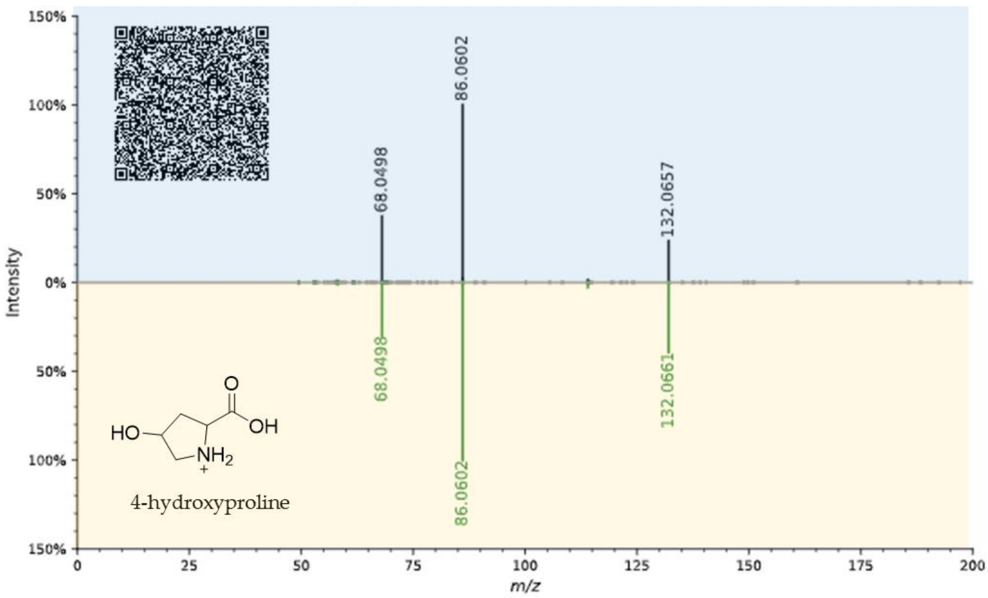

Figure S2.9. Comparison of MS/MS spectra of 4-hydroxyproline from *P. bermudensis* (top plot) and reference standard (bottom plot). Link could be found here, CCMSLIB00010013001

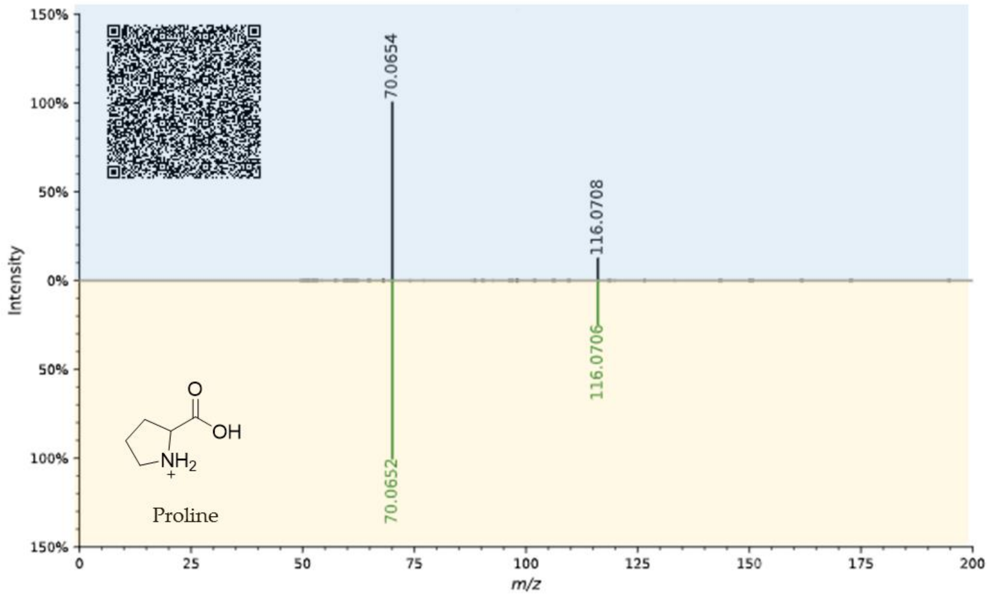

Figure S2.10. Comparison of MS/MS spectra of proline from *P. bermudensis* (top plot) and reference standard (bottom plot). Link could be found here, CCMSLIB00008851522

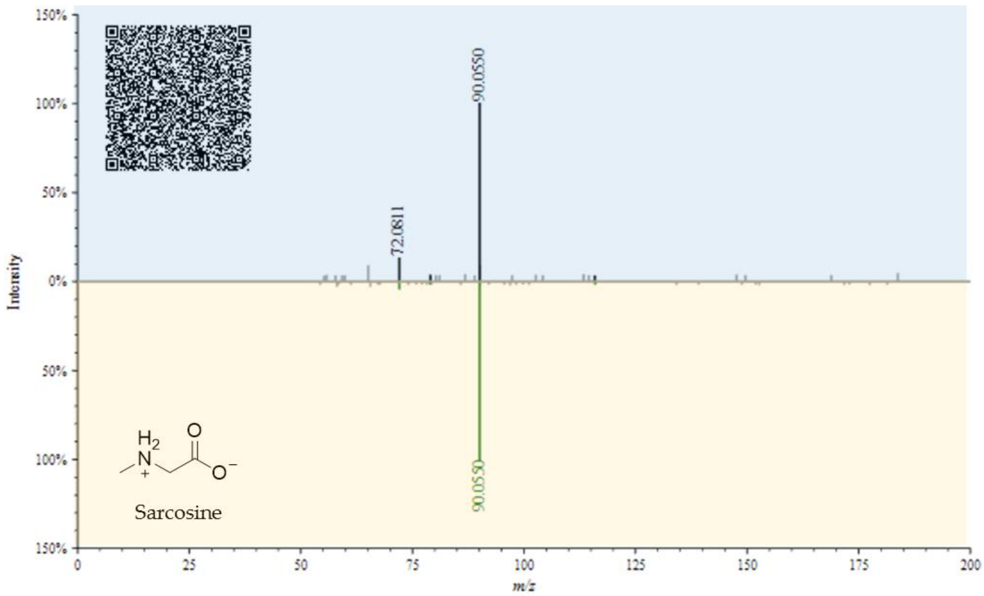

Figure S2.11. Comparison of MS/MS spectra of sarcosine from *P. bermudensis* (top plot) and reference standard (bottom plot). Link could be found here, CCMSLIB00008851520

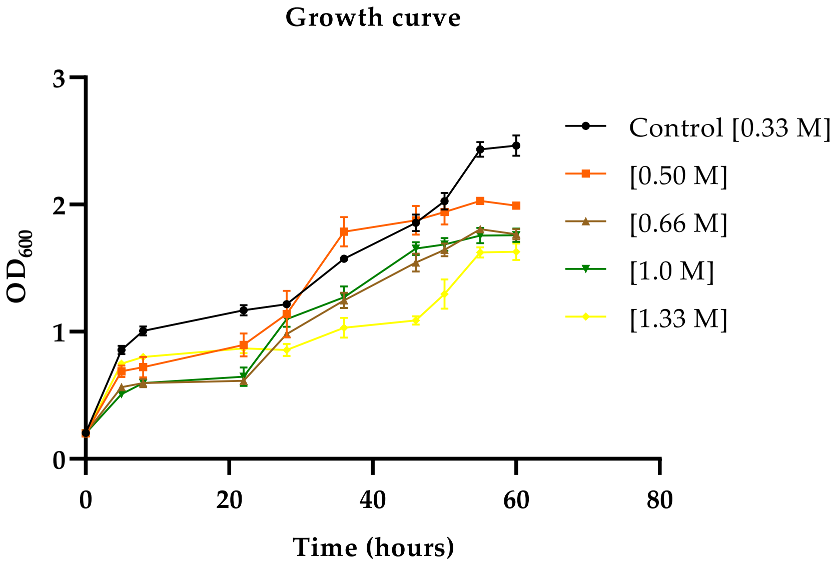

Figure S3.1. OD_{600} during the growth curve of *P. bermudensis* cultures grown in marine broth in NaCl concentration of 0.33 M – 1.33 M. Error bars represent standard deviation of three biological replicates. Sampling for extraction was taken at the late exponential phase.

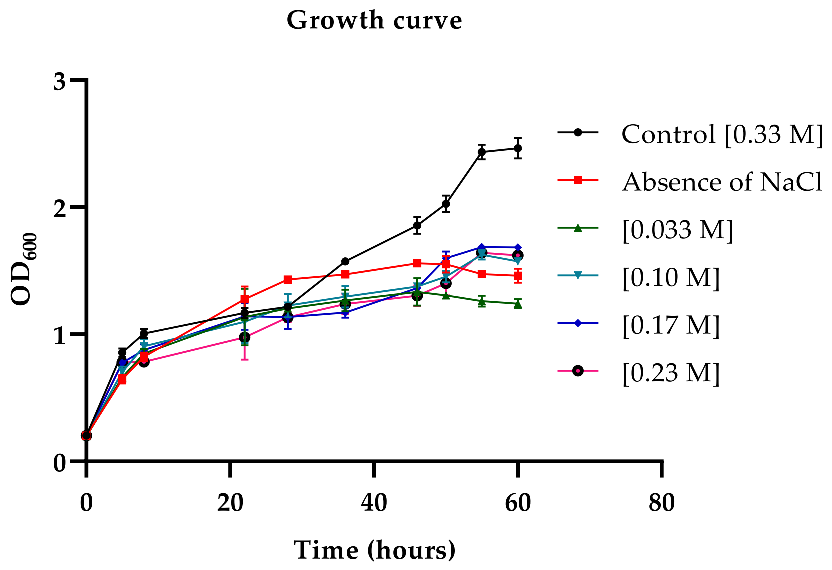

Figure S3.2. OD_{600} during the growth curve of *P. bermudensis* cultures grown in marine broth with NaCl concentration of 0.033 M – 0.33 M and the absence of NaCl. Error bars represent standard deviation of three biological replicates.

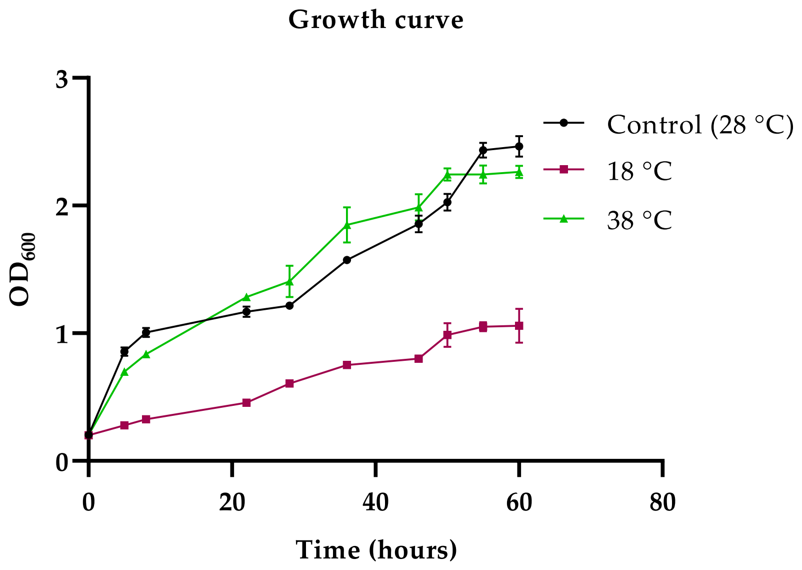

Figure S3.3. OD_{600} during the growth curve of *P. bermudensis* cultures grown in marine broth with a temperature range of 18 ℃ - 38 ℃. Error bars represent standard deviation of three biological replicates.

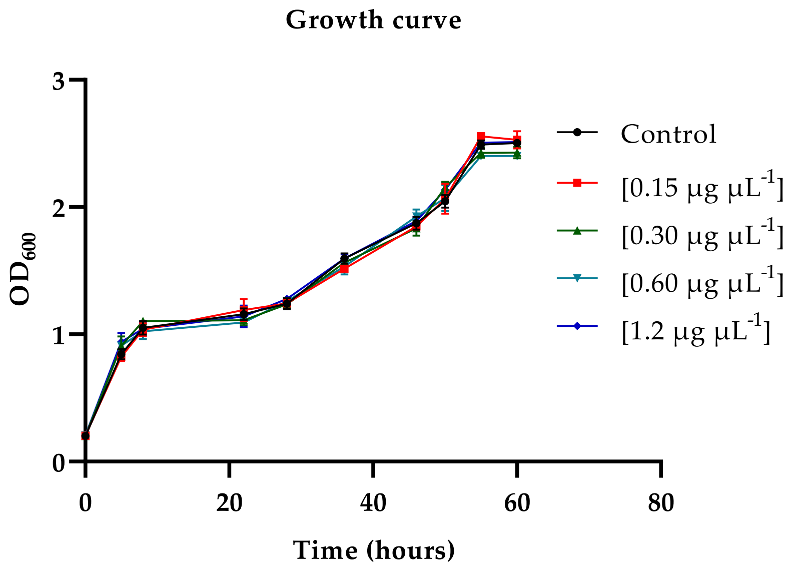

Figure S3.4. OD_{600} during the growth curve of *P. bermudensis* cultures grown in marine broth under the exposure to *T. striata extract* in different concentrations (0.15 μg μL^{-1}, 0.30 μg μL$^{-1,}$ 0.60 μg μL^{-1}, and 1.2 μg μL^{-1}). Error bars represent standard deviation of three biological replicates.

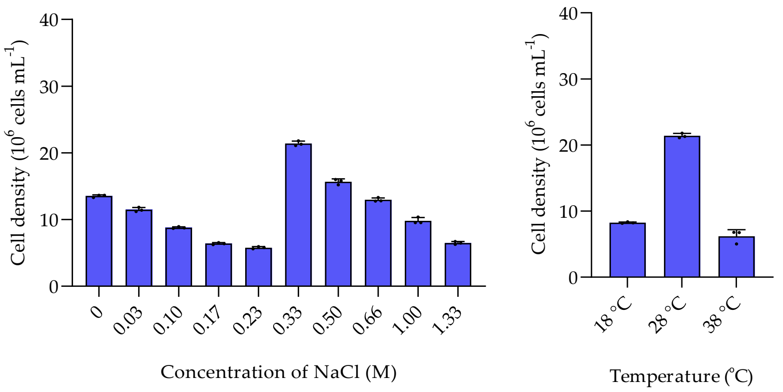

Figure S4. Cell densities of *P. bermudensis* under different stress treatments at the late exponential phase.

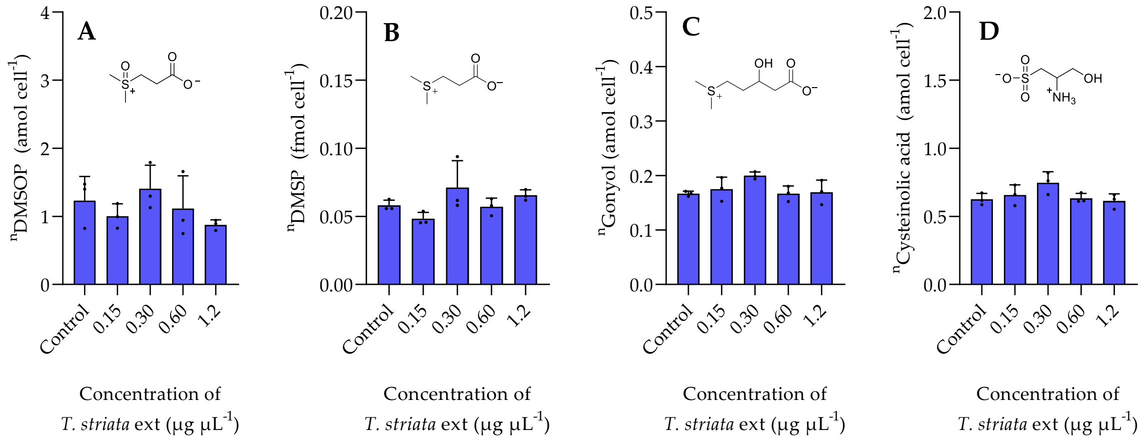

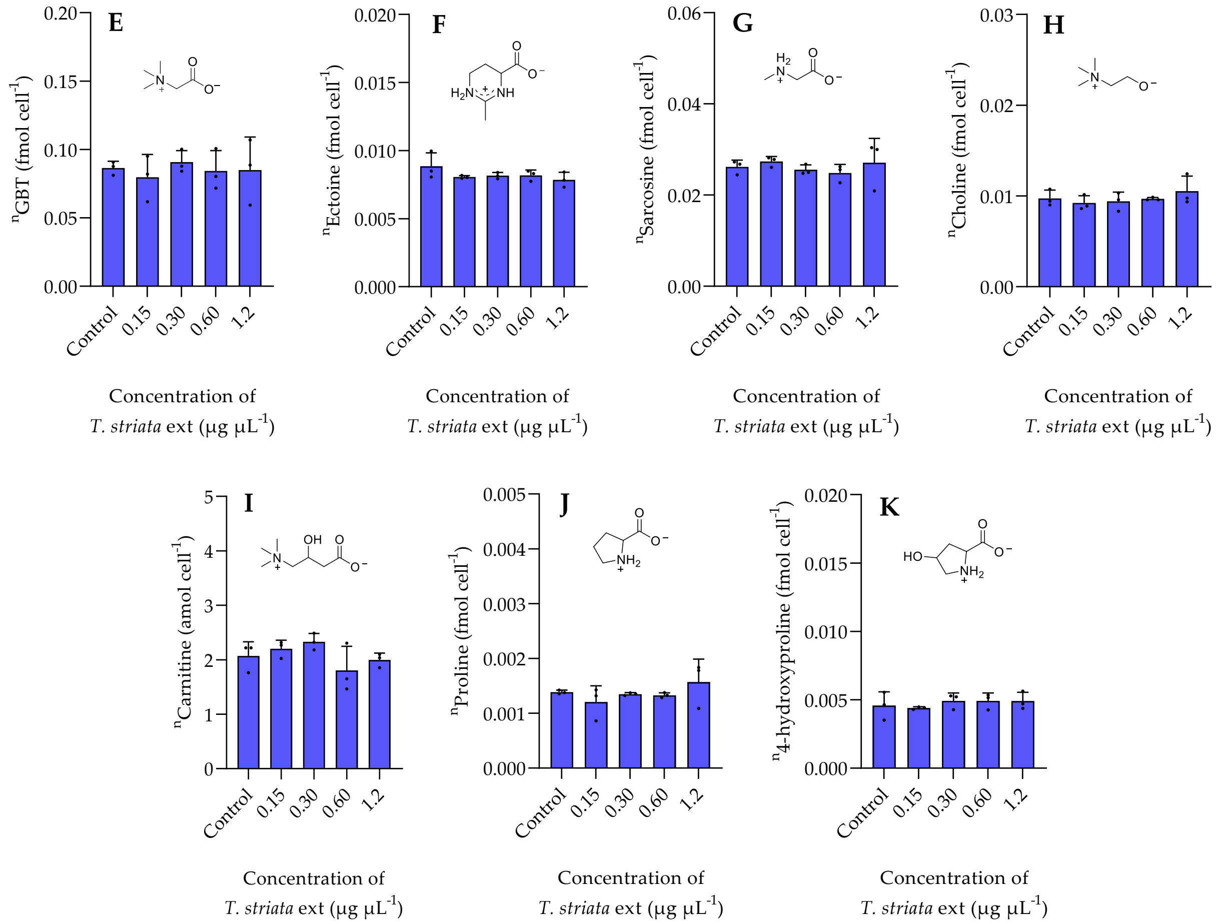

Figure S5. The intracellular concentration of sulfur- and nitrogen-containing zwitterionic metabolites in *P. bermudensis* HTCC2601 after treatment with *T. striata* extracts in different concentrations. (**A**) DMSOP, (**B**) DMSP, (**C**) gonyol, (**D**) cysteinolic acid, (**E**) GBT, (**F**) ectoine, (**G**) sarcosine, (**H**) choline, (**I**) carnitine, (**J**) proline, and (**K**) 4-hydroxyproline. Concentrations are normalized based on the cell count, error bars represent standard deviation (biological replicates, N = 3). Statistical analysis is based on One-Way ANOVA with a Tukey test for multiple comparison procedures. All statistical analyses were performed with a 95% confidence interval using GraphPad Prism 9.3.1 version. $p > 0.05$ is considered not significantly different. No statistically significant changes upon addition of *T. striata* extract were observed. Ext: extract; ^{n} = amounts per cell.

Table S1. Concentration of sulfur- and nitrogen-containing zwitterionic metabolites from *T. striata* by LC-HRMS

**Zwitterionic metabolites**	**Concentration of metabolites*** **(Mean ± Standard Deviation)**
Glycine betaine (GBT)	0.98 ± 0.11
DMSP	0.19 ± 0.013
Cysteinolic acid	0.046 ± 0.0016
Ectoine	0.016 ± 0.00089
Sarcosine	0.010 ± 0.00036
DMSOP	0.0091 ± 0.0017
Proline	0.00068 ± 0.00012
Choline	0.00040 ± 0.000043
Hydroxyproline	0.00045 ± 0.000045
Carnitine	0.000039 ± 0.0000047

* Mean ± Standard Deviation were expressed in μg mg^{-1} dried algal extract (biological replicates, N = 3)

Reference:

1. Bittremieux, W.; Chen, C.; Dorrestein, P. C.; Schymanski, E. L.; Schulze, T.; Neumann, S.; Meier, R.; Rogers, S.; Wang, M. Universal MS/MS visualization and retrieval with the metabolomics spectrum resolver web service. *bioRxiv* **2020**.
